# Antimicrobial Peptides: Potential Application in Liver Cancer

**DOI:** 10.3389/fmicb.2019.01257

**Published:** 2019-06-05

**Authors:** Chunye Zhang, Ming Yang, Aaron C. Ericsson

**Affiliations:** ^1^Department of Veterinary Pathobiology, University of Missouri, Columbia, MO, United States; ^2^Department of Surgery, University of Missouri, Columbia, MO, United States; ^3^Ellis Fischel Cancer Center, University of Missouri, Columbia, MO, United States; ^4^University of Missouri Metagenomics Center, University of Missouri, Columbia, MO, United States

**Keywords:** antimicrobial peptide, anticancer peptide, hepatocellular cancer, mechanism, design, nanoparticles

## Abstract

The physicochemical properties of antimicrobial peptides (AMPs) including size, net charge, amphipathic structure, hydrophobicity, and mode-of-action together determine their broad-spectrum activities against bacteria, fungi, protozoa, and viruses. Recent studies show that some AMPs have both antimicrobial and anticancer activities, suggesting a new strategy for cancer therapy. Hepatocellular carcinoma (HCC), the primary liver cancer, is a leading cause of cancer mortality worldwide, and lacks effective treatment. Anticancer peptides (ACPs) derived from AMPs or natural resources could be applied to combat HCC directly or as a synergistic treatment. However, the number of known ACPs is low compared to the number of antibacterial and antifungal peptides, and very few of them can be applied clinically for HCC treatment. In this review, we first summarize recent studies related to ACPs for HCC, followed by a description of potential modes-of-action including direct killing, anti-inflammation, immune modulation, and enhanced wound healing. We then describe the structures of AMPs and methods to design and modify these peptides to improve their anticancer efficacy. Finally, we explore the potential application of ACPs as vaccines or nanoparticles for HCC treatment. Overall, ACPs display several attractive properties as therapeutic agents, including broad-spectrum anticancer activity, ease-of-design and modification, and low production costs. As this is an emerging and novel area of cancer therapy, additional studies are needed to identify existing candidate AMPs with ACP activity, and assess their anticancer activity and specificity, and immunomodulatory effects, using *in vitro*, *in silico*, and *in vivo* approaches.

## Introduction

Antimicrobial peptides (AMPs), also known as host defense peptides (HDPs), exist in almost all species of vertebrates, invertebrates, and plants (Zasloff, 2002; Yang et al., 2016). Most AMPs consist of less than 100 amino acid residues, and they share several common features including cationicity, hydrophobicity, and amphipathic structure (Brogden, 2005; Zhang and Sunkara, 2014). They are constitutively or inducibly expressed in various tissues and organs that are constantly exposed to microbial pathogens (Huttner and Bevins, 1999), such as epithelial cells of skin and the gastrointestinal and respiratory tracts. AMPs possess broad-spectrum antimicrobial activities against bacteria, fungi, protozoa, and viruses (Ganz, 2003), and their application for treating multiple-drug resistant (MDR) pathogens are well-studied (McKelvey et al., 2014; Zhao et al., 2014; Yang et al., 2015; Maitz et al., 2018).

The antimicrobial functions of cationic AMPs can be categorized into two major mechanisms: (1) disrupting microbial membrane integrity via interactions with negatively charged components, and (2) inhibiting the function and synthesis of intracellular DNA, RNA, and protein (Bahar and Ren, 2013). In addition to their direct antimicrobial activity, AMPs have immunomodulatory properties, such as chemotactic activity to immune cells (Yang et al., 1999) and lipopolysaccharides (LPS)-neutralizing ability (Yang et al., 2016). The dual functions of AMPs reduce the ability of microbes to develop resistance to these peptides. Several recent studies show that AMPs also have anticancer activity (Hoskin and Ramamoorthy, 2008; Baindara et al., 2017; Baindara et al., 2018), and peptides with both antimicrobial and anticancer activities have been reviewed previously (Felício et al., 2017). Based on the molecular characteristics and observed properties of AMPs, selective anticancer peptides (ACPs) could be identified or designed for use as novel therapeutic agents for cancer treatment.

Hepatocellular carcinoma (HCC) is a leading cause of malignant cancer death worldwide (Waghray et al., 2015). The major risk factors contributing to HCC are infection with hepatitis B or C viruses, abuse of alcohol, intake of microbial metabolite aflatoxin B1, and non-alcohol fatty liver disease (NAFLD) (Llovet et al., 2016). Despite advances in diagnosis, mortality associated with HCC continually rises due to the lack of effective therapies. Thus, novel HCC treatment strategies are urgently needed (Balogh et al., 2016).

AMPs with dual antimicrobial and anticancer activities (i.e., ACPs) are promising therapeutic agents, which can be used to combat HCC as a stand-alone treatment, or as part of a synergistic treatment regimen. In this review, we will summarize the new candidate peptides for HCC treatment, the potential mechanisms of action of ACPs against HCC, the design and modification of anti-HCC peptides, and the strategies to promote their application.

## Peptides With Anti-HCC Activity

In the past few years (i.e., from 2012 to 2019), an increasing number of ACPs has been evaluated or designed for HCC treatment (Gaspar et al., 2013). The sequences, sources, and functions of new candidate peptides have been summarized in Table 1. In summary, anti-HCC peptides can be derived from bacteria, marine and terrestrial animals. They can be identified among superficially binding peptides using phage-displayed selection on HCC cancer cell lines. These ACPs can target ion channels, phospholipids, or molecules in specific signaling pathways to induce cancer cell apoptosis.

**TABLE 1:** Summary of anti-HCC peptides and their characteristics.

**Peptide**	**Sequence**	**Description**	**Function**	**References**
Tv1	SEQUENCETRICCGCY WNGSKDVCSQSCC	A venom peptide from marine terebrid snail, *Terebra variegata*	Tv1 inhibits the proliferation of murine HCC cells by down-regulation of the cyclooxygenase-2 (COX-2) pathway, and induces cell apoptosis in a Ca^2+^-dependent manner.	Anand et al.,2019
FFW	RRKFA KFQWI	An inhibitor targeting Sal-like 4 (SALL4)-nucleosome remodeling deacetylase (NuRD) complex	FFW shows proapoptotic and antimigration effects in SALL4-expressing hepatocellular carcinoma (HCC) cells.	Liu et al.,2018
SP94	SFSIIH TPILPL	A peptide, isolated using phage-displayed selection, specifically targeting human HCC cell lines (e.g., Mahlavu and SK-HEP-1)	SP94-conjugated, doxorubicin-encapsulated liposomes enhance HCC apoptosis and decrease tumor angiogenesis.	Wu et al.,2018
R-Tf-D-LP4	KWTWKNSNGATWALNVATE LKKEWTWSHRPYIAH	A cell-penetrating peptide (CPP) derived from a mitochondrial multifunctional protein in the voltage-dependent anion channel (VDAC1)	It induces liver cancer-derived cell apoptosis *in vitro* and inhibits tumor growth in three different liver cancer mouse models: diethylnitrosamine (DEN)-induced HCC, metabolically high-fat diet-induced HCC, and a subcutaneous HepG2 cell xenograft model.	Pittala et al.,2018
GG-8-6	Cyclo-VLPILLVL	A cyclopeptide derived from the lead compound Grifficyclocin B from plants of *Goniothalamus* species	GG-8-6 (1) has IC50 values of 6.38 μM and 12.22 μM against SMMC-7721 and HepG2, respectively. GG-8-6 (1) also induces apoptosis and G2/M arrest of HCC cells, probably through the activation of caspase pathways.	Chen et al.,2018
BR2	RAGLQFP VGRLLRRLLR	A nonspecific cell-penetrating ACP derived from buforin IIb	BR2-modified liposomes loaded with cantharidin, the active compound isolated from Chinese medicine blister beetles, significantly increases anti-HCC efficacy.	Zhang et al.,2017
β3	DLYYLMDLSYSMKGGDLYYL MDLSYSMKGGDLYYLMDLSYSMK	A trimer peptide of anti-adhesion peptide β (DLYYLMDLSYSMK)	β3 peptide shows anti-adhesion activity of highly metastatic HCC cell line HCCLM6 to fibronectin (FN) and inhibits HCC recurrence *in vivo* and prolongs the survival time of HCC nude mice LCI-D20 following hepatectomy.	Wang et al.,2016
CecropinXJ	RWKIFKKIEKMGRNI RDGIVKAGPAIEVLGSAKAIGK	A cationic antimicrobial peptide (AMP) originally isolated from the larvae of *Bombyx mori*	CecropinXJ induces S cell cycle arrest and apoptosis of human HCC cell line Huh-7 cells by activating caspase-3 and poly (ADP-ribose) polymerase.	Xia et al.,2016
GW-H1	GYNYAKKLA NLAKKPANALW	A novel cationic amphipathic AMP GW-H1	GW-H1 induces caspase-dependent apoptosis of HCC cell lines including J5, Huh7, and Hep3B. GW-H1 also suppresses J5-xenografted HCC development in nude mice in a dose-dependent manner.	Chen et al.,2012
Bovicin HC5	VGXRYASX PGXSWKYVXF	A bacteriocin from *Streptococcus bovis* HC5	It shows direct killing. The IC 50 of Bovicin HC5 is 289.30 μM for HepG2 cells (human HCC cell line).	Paiva et al.,2012

## Mechanisms of ACPs Against HCC

In addition to the abovementioned functions of ACPs, peptides derived from AMPs or natural sources may have the following capacities to treat HCC: direct killing, anti-inflammation, immune modulation, and wound healing. The following four paragraphs will describe the details of their killing mechanisms and relevant examples.

### Direct Killing Activity

**FIGURE 1:**
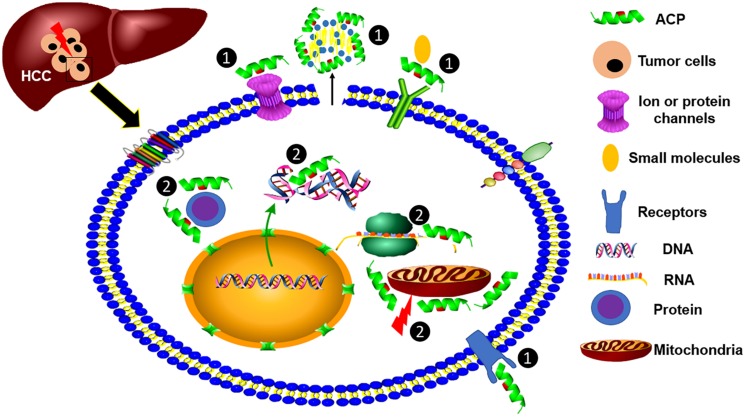
Modes-of-action of anticancer peptides (ACPs). ACPs show killing efficacy against HCC cells through two modes-of-action, including ① targeting cell surface molecules, such as specifically binding to a receptor and nonspecifically binding to negatively charged phospholipids, and ② binding with intracellular cell organelles or RNA, DNA, and proteins to kill cancer cells.

The antimicrobial activity of AMPs is elicited by the electrostatic interaction between the cationic peptides and the negatively charged bacterial components, such as LPS and lipoteichoic acid (LTA), and is followed by the insertion into, and interruption of, the microbial membrane (Yang et al., 2018). Notably, the anionic phospholipid components of cancer cell membranes are different from normal cells. The density of negatively charged phosphatidylserine (PS) in the cancer cell membrane is higher than that of normal cells (Utsugi et al., 1991), making them more sensitive to ACPs. For example, an enantiomeric 9-mer peptide derived from beetle defensin exhibited more selective cytotoxicity to mouse myeloma cells (P3-X63-Ag8.653) than normal leukocytes (Iwasaki et al., 2009). The mode-of-action was suggested by the strong correlation to the density of negatively charged phosphatidylserine in the myeloma cell membrane, as well as several other cancer cell lines. After initial binding, peptides then form pores in the cancer cell membrane to cause apoptotic or necrotic cell death. In addition, another group of ACPs are tumor-targeting peptides (TTPs) which can specifically bind cancer cell surface markers (e.g., arginine/glycine/aspartic acid motifs) (Boohaker et al., 2012), and these surface-associated molecules are commonly overexpressed on tumor cells. Furthermore, cancer cell membranes often contain more microvilli per surface area, which further enhances the binding of ACPs to cancer cells to increase their anticancer efficacy (Deslouches and Di, 2017). In Figure 1, we summarize the potential mechanisms of ACP activity against HCC cells.

### Anti-inflammatory Activity

When gut permeability is compromised, gut microbiota and their products including endotoxins and flagellin may disseminate directly from intestine to liver via the portal vein to induce hepatic inflammatory responses, which may ultimately lead to fibrosis, cirrhosis, and HCC. Toll-like receptor (TLR) signaling pathways have been implicated in the inflammatory reactions during the development of liver cancer. TLR4 is an extracellular pathogen recognition receptor which binds LPS, and plays a vital role during the chronic inflammation in HCC (Sepehri et al., 2017). The expression of inflammatory molecule Hepcidin can be induced by LPS via hepatocyte TLR4-mediated signaling pathways (Lee et al., 2017). TLR5 in hepatocytes protects against high-fat diet-induced liver disease via binding of bacterial flagellin (Etienne-Mesmin et al., 2016). ACPs derived from AMPs could take advantage of their strong electrostatic interactions with the negatively charged LPS of Gram-negative bacteria (Yang et al., 2016), LTA of Gram-positive bacteria (Gustafsson et al., 2010), and flagellin in both groups, when used as anti-inflammatory agents. In addition, TLRs are also widely expressed in a variety of liver immune cells, including macrophages, dendritic cells, T cells, and B cells. Therefore, modulation of TLR activity is able to elicit an anti-HCC activity in liver, and the peptide agonists of TLRs may serve as novel therapeutic agents for HCC treatment (Zou et al., 2016).

### Immune Modulation

Tumor-associated antigens (TAA) can be recognized by antigen-presenting cells and induce the activation of tumor-responsive T lymphocytes. It has been shown that TAA-derived long peptides can elicit Th_1_ T cells and cytotoxic T lymphocyte (CTL)-mediated anticancer immune responses through cross-presentation (Tomita and Nishimura, 2013). Alpha-fetoprotein (AFP) is a well-known TAA in HCC. HLA-A^*^0201-restricted peptides derived from human AFP stimulate specific T-cell responses both in cultured peripheral blood lymphocytes of healthy people and T-cells from patients (Butterfield et al., 2003). A large number of tumor-infiltrating lymphocytes (TILs) including CD4^+^ T cells in the tumor/liver interface and CD8^+^ T cells inside of the tumor have been demonstrated in HCC (Kasper et al., 2009). Thus, TAA-derived peptides might be particularly useful to enhance anti-HCC treatment. In addition, cell-mediated therapy using the adoptive transfer of TILs is considered as one of the immunotherapeutic strategies in cancer treatment (Geukes Foppen et al., 2015). However, cancer cells may evade the immune surveillance either by alteration of their antigen presentation or by secreting cytokines and chemokines which induce Treg cells to establish an immunosuppressive microenvironment (Nishida and Kudo, 2017). ACPs with immunostimulatory activity could be applied to increase the efficacy of cell-mediated therapy. For instance, Tyroserleutide is an immunostimulatory peptide comprising three amino acids (YSL), which can stimulate the antitumor effects of macrophages against human HCC cell line BEL-7402 (Zhi et al., 2006).

### Wound Healing Activity

Liver fibrosis is a wound healing process associated with chronic liver injury, which often leads to cirrhosis and HCC at the end-stage (Ramakrishna et al., 2013). AMPs as endogenous mediators enhance wound healing (Mangoni et al., 2016) through antimicrobial activity, LPS neutralization, angiogenesis, and chemotactic activity. For example, recombinant human AMP LL-37 promoted wound healing by inhibiting the activation of macrophages with LPS and by inducing the proliferation and migration of endothelial cells, vascularization, and re-epithelization (Ramos et al., 2011). In addition, natural AMPs can be modified or combined with other short peptides to improve their potential abilities. The novel Tylotion chimera peptide (Tylotion-sC18^*^), a covalently coupled peptide with a wound-healing promoting sequence (Tylotion) and a cell-penetrating peptide (CPP), showed promising wound-healing activity and antimicrobial activity (Horn and Neundorf, 2018). ACPs coupled with CCPs may also show similar functions in the treatment of liver injury during the development of HCC (Sanyal et al., 2010).

## Design of Anticancer Peptides (ACPs)

### Structure of Antimicrobial Peptides

Although variable in length, amino acid composition, and host origin, AMPs can be classified into the following four types based on their secondary structures (Powers and Hancock, 2003): linear or extended, α-helical, circularly looped, and β-sheeted peptides. The α-helical and β-sheeted structures are more frequently seen in natural peptides and their synthetic analogs. Regardless of their structure, common characteristics of these AMPs include their cationic and amphipathic nature, which accounts for their abilities to interact with the negatively charged components of bacterial cell walls, and amphipathic cell membranes (Yang et al., 2017). ACPs derived from AMPs or other natural peptides show similar structures as AMPs and their analogs (Wu et al., 2009).

### Modification of Anticancer Peptides

**FIGURE 2:**
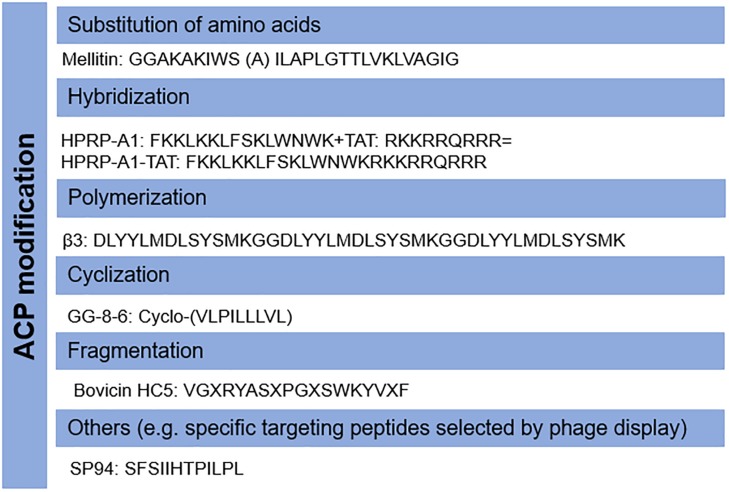
Modifications of anticancer peptides (ACPs). The structure and amino acid components of ACPs can be modified by several methods including the substitution of amino acids, hybridization, polymerization, fragmentation, cyclization, and others.

Host AMPs have also been shown to have anticancer ability, however, they may not be directly applicable as anticancer agents due to their low killing activity. The properties of candidate ACPs can be modified according to their mode-of-action. For example, the cytotoxic activity of cyclotides is dependent on targeting phosphatidylethanolamine phospholipids (Troeira Henriques et al., 2014). Therefore, novel analogs could be designed to increase the membrane-binding affinity and selectivity to cancer cells by modulating their amino acid components (Rady et al., 2017), as with AMPs (Yang et al., 2018). For instance, a modified peptide CB1a derived from the well-known AMP Cecropin B demonstrated promising activity against leukemia and carcinoma cells with low cytotoxicity to non-cancer cells (Wu et al., 2009). In addition, a variety of other strategies including cyclization (Chen et al., 2018), hybridization (Lim et al., 2013; Hao et al., 2015), polymerization (Wang et al., 2016), fragmentation (Li et al., 2006; Paiva et al., 2012), and others (Lo et al., 2008) can be applied to increase their efficacy and stability, and to decrease unwanted collateral cytotoxicity. The common modifications of ACPs have been shown in Figure 2. ACPs can also be designed and characterized prior to synthesis *in silico* to reduce time and labor (Kumar and Li, 2017).

## The Potential Application of ACPs

ACPs have dual killing mechanisms, either specifically/nonspecifically binding with targets, or modulating immune responses to kill cancer cells, making them attractive candidates for HCC treatment. To achieve peptide-based anticancer therapy, delivery systems including peptide-derived vaccines, nanoparticles, and liposomes need to be explored. Finding appropriate delivery systems will improve the efficacy of peptides for cancer treatment.

### Peptide-Based Vaccines

Peptide-based vaccines are commonly water-soluble, easy to store, and can be customized to target specific objectives and generated in a large scale. Glypican-3 (GPC3) of heparin sulfate proteoglycans, specifically overexpressed in HCC (>80%), is a promising cancer immunotherapeutic target. GPC3-derived peptide vaccines have been shown to induce an increase in peptide-specific CTLs (Iwama et al., 2016) and are being evaluated in clinical trials (Nobuoka et al., 2013). However, there are some limitations for peptide vaccines, such as poor immunogenicity, and low efficacy and stability in physiological conditions. Several strategies have been applied to overcome these issues, such as the use of immunostimulatory adjuvants, new delivery systems with nano- or micro-particles, and multi-epitope approaches (Skwarczynski and Toth, 2016). AMPs with anticancer property can also be used directly as adjuvants for vaccines. For instance, immunization of AMP GE33 with inactivated murine bladder carcinoma (MBT-2) cells induced more MBT-2-specific tumor antigens and CTLs and NK cells in mice, accompanied by a decrease of the expression of VEGF (Huang et al., 2014). Clinical trials for peptide-based vaccines in cancer therapy for targeting TAAs have been reviewed recently by Bezu et al. (2018) group. Targeting tumor-specific mutated antigens (e.g., β-catenin and ERBB2IP) can minimize the off-tumor cytoxicity and advance more personalized treatments (Ilyas and Yang, 2015). Several peptide vaccines have demonstrated beneficial immune effects in clinical trials (Boohaker et al., 2012). For example, vaccine MKC-1106-PP co-targeting preferentially expressed antigen in melanoma (PRAME) and prostate-specific membrane antigen (PSMA) could induce antigen-specific T cells in patients with solid tumors in a phase I study (Weber et al., 2011).

### Nanoparticles

Nanocarriers such as gold nanoparticles and liposomes have been broadly applied in the delivery system due to a long drug half-life, bioactivity, and cell selectivity (Zhao et al., 2018). Delivering ACPs by nanocarriers is another promising strategy for cancer therapy. Wu et al. (2018) reported that the use of liposomes to deliver HCC-targeting peptide SP94 (SFSIIHTPILPL), selected by phage-display, enhances their therapeutic efficiency in a mouse HCC xenograft model and increases its distribution in tumor tissues. Currently, more studies are needed to identify unique true predicted neoantigens (TPNAs) as potential candidates as immunotherapeutic agents for HCC (Petrizzo et al., 2018).

## Summary

HCC remains a major cause of cancer-realted mortality without effective treatment options. Thus, alternative therapeutic agents are urgently needed for HCC therapy. ACPs offer great potential for liver cancer therapy with a variety of advantages, such as broad anticancer spectrum, ease of design and modification, and low production costs. Incorporating ACPs in a drug delivery system can improve their anticancer efficacy, target specificity, and half-life time. Compared to conventional chemotherapy, ACPs exert less systemic effect and cancer cells develop negligible resistance to these peptides. In addition, ACPs could be applied as a synergistic strategy with other therapies including hormonal therapy (e.g., Tamoxifen), biochemical therapy (e.g., IFN-γ), and chemotherapy (e.g., Sorafenib) (Shaaban et al., 2014). The primary challenges in the development of clinically useful are associated with their onsite delivery and off-target binding. Similar to AMPs, ACPs have potential unwanted cytoxicity to non-cancerous cells adjacent to a tumor, and immune cells in the tumor environment. Thus specificity and off-site cytoxicity are two active areas of investigation in the development and application of ACPs. Currently, the number of candidate ACPs is much lower compared to the number of antibacterial peptides and antifungal peptides (Shoombuatong et al., 2018). There are no specific criteria for the design and modification of ACPs (Gaspar et al., 2013), and ideal ACPs might best be designed on the basis of specific tumor microenvironments to optimize the stability and selectivity via manipulation of their sequences, net charges, amphipathic structures, and hydrophobicity. Such structure-activity relationship investigations are required to design new ACPs (and modify existing ACPs), and to test their efficacy and pharmacokinetic properties *in vivo* animal models. We hope that the peptide examples listed in this review will provide some clues or inspiration for researchers in the development of new ACPs for the treatment of HCC.

## Author Contributions

CZ wrote the Introduction and Summary. MY and AE drafted the remaining manuscript and revised the manuscript.

## Conflict of Interest Statement

The authors declare that the research was conducted in the absence of any commercial or financial relationships that could be construed as a potential conflict of interest.

## Abbreviations

ACP: anticancer peptide
AMP: antimicrobial peptide
CPP: cell-penetrating peptide
GPC3: Glypican-3
HCC: hepatocellular cancer
HDP: host defense peptide
LPS: lipopolysaccharide
LTA: lipoteichoic acid
MBT-2: murine bladder carcinoma
MDR: multiple-drug resistant
NAFLD: non-alcohol fatty liver disease
PRAME: preferentially expressed antigen in melanoma
PSMA: prostate-specific membrane antigen
TAA: Tumor-associated antigens
TIL: tumor-infiltrating lymphocyte
TLR: Toll-like receptor
TTP: tumor targeting peptide.
